# Incidental Radiological Finding of an Intravenous Needle in a Pediatric Cervical Spine

**DOI:** 10.1155/crpe/9950731

**Published:** 2024-11-27

**Authors:** Thana Namer, Rahaf Alanazi, Maryam Al Karawi, Mahfood Saeed, Sarmad Al Karawi

**Affiliations:** ^1^Division of Neurosurgery, Department of Surgery, King Abdulaziz Medical City, Ministry of National Guard-Health Affairs, Riyadh, Saudi Arabia; ^2^College of Medicine, King Saud bin Abdulaziz University for Health Sciences, Riyadh, Saudi Arabia; ^3^King Abdullah International Medical Research Center, Riyadh, Saudi Arabia; ^4^College of Medicine, James Cook University, Townsville, Australia; ^5^Division of Neurosrugery, Department of Surgery, Al Hammadi Hospital, Riyadh, Saudi Arabia

## Abstract

Cases of cervical foreign bodies are considered rare, and cases of needle that have migrated into the spinal canal are fairly uncommon. The most well-documented cases are those of acupuncture needles. We present a case of an incidental finding of an intravenous needle extending posteriorly between C7 and T1 interspinous space and ending at the level of C5-C6 interspace in a 2-year-old boy. We discuss the possible port of entry and the management of such findings.

## 1. Introduction

Intraspinal foreign bodies typically result from penetrating traumatic injuries, including firearms, assault, and accidents. These incidents can also be iatrogenic when surgical objects are accidently left inside the body or intentionally used as a means of treatment. In most cases, there is a clear history of the event [1]. In the literature, most reports involve the findings of acupuncture needles or charm needles in the spine [2]. In most of these cases, the needles were found as a result of a penetrating injury when the foreign body was forcefully inserted posteriorly or anteriorly [3]. Few other articles reported findings of a sewing/knitting needle in the spine after a fall or a stab [4]. Although unlikely, a transesophageal route could be considered with the migration of a swallowed needle to the vertebral column [3]. In some of these cases, the needles in the spinal canal were discovered to be the source of pain or neurological symptoms decades after the initial injury while others are discovered incidentally [5]. Nevertheless, when penetrating cervical spine injury is suspected, child abuse should be considered. Herein, we report an unusual case of an incidentally detected asymptomatic spinal IV needle in the cervical spine of a 2-year-old boy. We discuss the possible port of entry and the management of such findings.

## 2. Case Presentation

### 2.1. History and Examination

**Figure 1:**
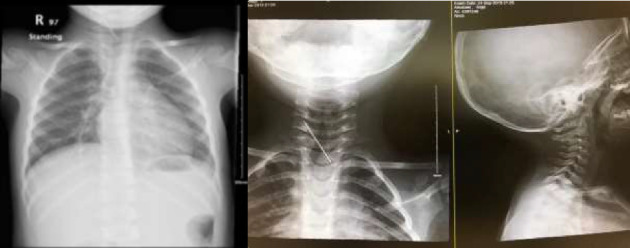
Chest X-ray showing a 4 cm, hyperdense foreign body projecting over the posterior lower neck.

A 2 year-old-girl was brought to the emergency department by her parents for tuberculosis screening as it was positive in her family members. She was referred to our care after chest X-ray findings of a foreign body in her neck (Figure 1). Upon evaluation, the patient denied any symptoms of radiculopathy, tingling, weakness, or bladder and bowel issues. She did not report difficulty with secretions or drooling. She denied history of accidental falls or intentional ingestion. At presentation, the child was conscious, alerted, and oriented. She was ambulating with normal gait. The cranial examination was unremarkable. Muscle strength, tone, and deep tendon reflexes were normal and meningeal signs were negative. There were no signs of any puncture, laceration, or redness over the skin on back or front of the neck. Suspected Child Abuse and Neglect (SCAN) Team was contacted and this case was reported to the relevant authority for further criminal investigation. SCAN team ruled out any signs of child abuse or neglect. The parents reported leaving the child for 2 weeks in her grandparents' home in order to run few errands with the presence kids around her age, a housemaid, and her uncle who is a paramedic.

### 2.2. Investigation

**Figure 2:**
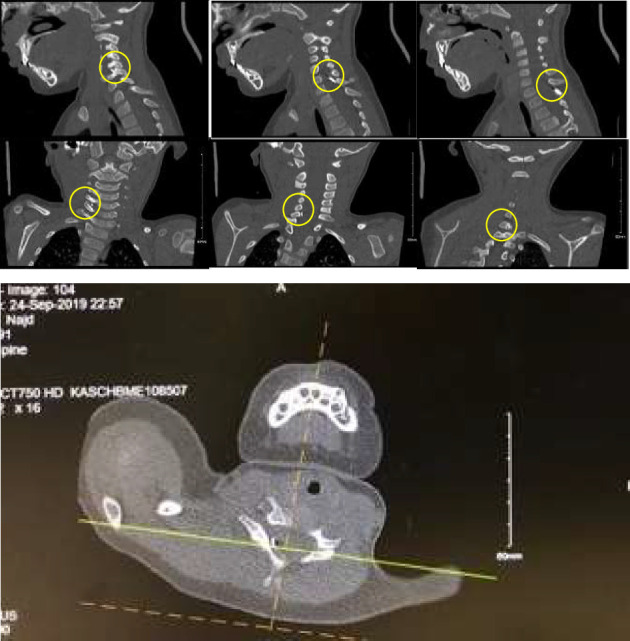
CT cervical with sagittal, coronal, and axial views showing an obliquely oriented hyperdense linear foreign body, likely a needle, measuring about 4 cm and starting from C7-T1 interspinous space close to the left lamina of T1 extending superiorly to the right and ending at the C5-C6 level, abutting the C5-C6 exiting right nerve root as well as the foraminal segment of the right vertebral artery (VA).

**Figure 3:**
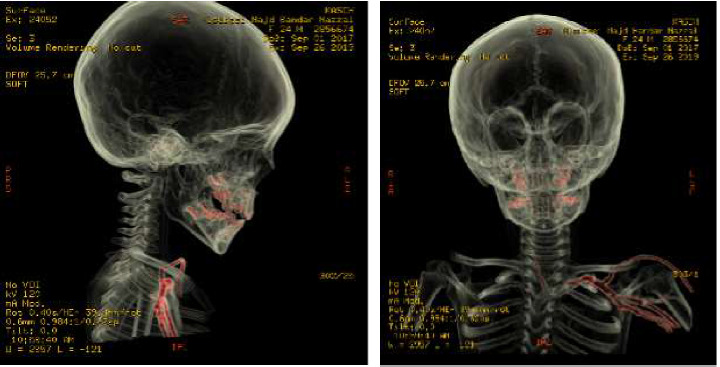
3D from CT angiography with coronal and sagittal views showing bilateral vertebral arteries are normally opacified with redemonstration of intraspinal needle extending from C7-T 1 up to C5-C6. The needle is abutting the right vertebral artery and the anterior spinal artery at the levels of C5 and C6 with no evidence of vascular injury.

As the mechanism of injury was not known, we wanted to rule out spinal cord injury or other vascular injuries. Therefore, we requested cervical spine computed tomography (CT), CT angiogram, and skeletal survey. A CT Angiogram were performed to confirm the X-ray findings which demonstrated a 4 cm needle-shaped object entering between C7 and T1 interspinous space and ending at the level of C5-C6 interspace (Figure 2), passing medial to the vertebral artery with no violation of the vertebral artery (Figure 3). MRI scan was not obtained in order to avoid further injury. Abdominal and chest X-rays revealed no other similar foreign bodies and the skeletal survey was negative of any fractures.

### 2.3. Surgery and Clinical Outcomes

**Figure 4:**
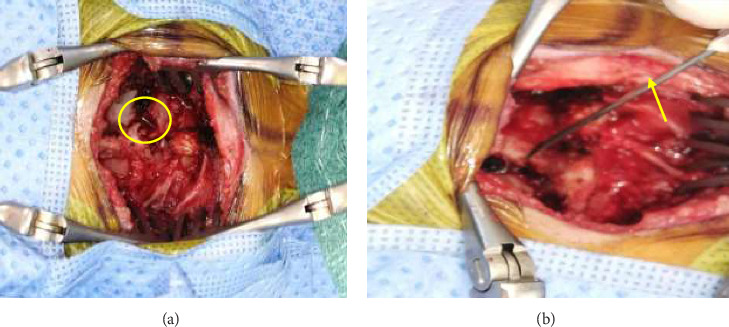
(a) Intraoperative finding of 22-gauge broken needle in cervical spine canal measuring about 4 cm, (b) needle after removal.

**Figure 5:**
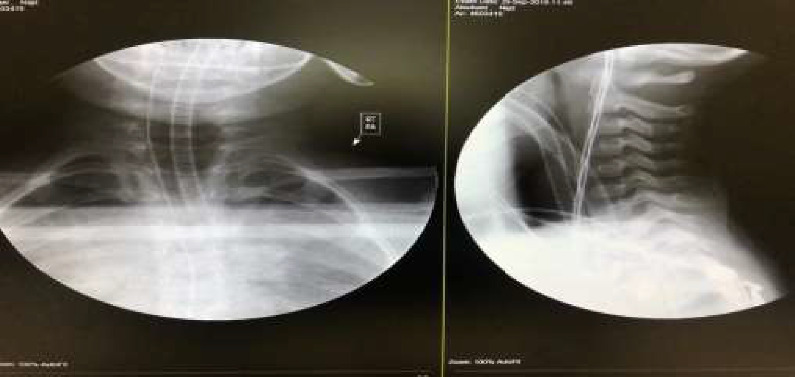
Intraoperative cervical X-ray showing complete removal of the foreign body.

The decision was made to surgically remove the needle because it has penetrated into the foramen transversarium and was in contact with wall of the cervical dura mater passing near the right vertebral artery which could lead to potential damage to the spinal cord and vertebral artery. Therefore, the patient underwent a posterior right-sided T1 hemi-laminotomy with removal of the foreign body. With fluoroscopy guidance, we confirmed the correct level. Then About 6 cm long skin incision was made in the midline, followed by subfascial dissection. The tip of the needle was identified and confirmed with the use of the X-ray. The operative microscope was brought in to explore the field for the foreign body. The foreign body was visualized and easily extracted after performing a right-sided laminotomy around the needle enters point using a diamond drill. There was no cerebrospinal fluid (CSF) leak after the removal of the foreign body. The defect was covered by fibrin glue (Tisseel). The foreign object was found to be a broken syringe needle that had the same size of a 22-gauge needle (Figure 4). Intraoperative X-ray was done to confirm complete removal of the needle (Figure 5). Hemostasis was achieved and the wound was closed. Before the final closure, the circulating nurse announced that all counts were correct and complete including needle, sponge, and instrument.

### 2.4. Postoperative Course

Postoperatively, CTA was performed, which confirmed that there was no arterial injury. The patient followed up after 2 months, she appeared playful and active, of normal development and no neurological deficits. On inspection of the wound, it healed appropriately and appeared dry and clean.

## 3. Discussion

Intraspinal foreign bodies in patients with few or no symptoms have rarely been reported, and cases of needles in the cervical spine that have migrated into the spinal canal are quite uncommon. Differential diagnoses include acupuncture needles, charms, sewing needles, intravenous drug use needles, dental restorative instruments, root canal fillings, and deliberately implanted postsurgical material [2].

Limited reports of cervical spine needles have been documented in the literature (Table 1). In one case [6], the patient had been performing acupuncture on herself in the posterior neck region. Similarly, one patient had received an acupuncture-like procedure, performed by a nonmedical practitioner, in his back region for chronic dizziness and neck stiffness [7]. There are three case reports of sewing needles found in the cervical spine. In one case, the sewing needle accidentally penetrated the cervical spine when the patient fell during a soccer game [4]. Furthermore, two articles [8] reported cases where patients stabbed themselves in the posterior region of their necks with sewing needles.

Unlike previously reported cases, in our case, the needle was found to be a broken 22-gauge intravenous syringe needle. Generally, acupuncture needles range in length from 7 to 200 mm, while IV needle sizes can vary. Sewing needles are straight with one tip having a single hole, are thicker than acupuncture needles, and are made of iron. If left in place for a long time, they could erode, leading to breakage and infection [2]. To the best of our knowledge, this is the only case report of an incidental IV needle in the cervical spine of a child.

Considering other potential ports of entry, Persad and Vitali [3] reported a case of a 17-year-old girl with a migration of a swallowed needle to the cervical spine. In our case, the port of entry is unknown as the patient denied any symptoms and there were no wounds, scars, or lacerations indicating any site of entry. Therefore, the findings could be the result of an old penetrating injury with a healed wound, or the needle may have migrated through the transesophageal route, similar to what has been previously reported by Persad et al. The transesophageal entry of the foreign body may present challenges in terms of infection risk, and intubation and antibiotic prophylaxis are recommended in accordance with prior reports [3].

In the present case, the needle extended between the C7 and T1 interspinous space and the level of the C5-C6 interspace (Figure 2), abutting the C5-C6 exiting right nerve and passing medial to the vertebral artery without violating the VA. These findings are similar to previously reported articles. In a few cases, the needle entered posteriorly and ran anterolateraly between the dura and the VA [6–8]. In two other cases, the needle ran anteriorly and entered the neural foramen, passing medial to the VA [3, 4].

Fumihiro et al. [8] reported CSF leakage after the removal of the needle, which was repaired by suturing the ligamentum nuchae tightly. This complication could be attributed to the force of insertion, the size and location of the object, or the length of time the object was in situ, as the needle was inserted 2 years prior to presentation, which would result in local inflammation and scarring around the foreign body [3]. Fortunately, in our case, the patient did not experience any complications after surgery.

The clinical symptomatology of these cases in the literature has been variable, ranging from being completely asymptomatic to CSF fistulas to severe motor and sensory deficits [4]. Anderson et al. [9] reported a case of spinal cord injury with a decrease in left-arm power (4/5) and altered sensation over the left C5–C8 dermatomes. In our case, the patient presented with no neurological signs or symptoms, and the needle was found incidentally. Given this scenario, it is vital to obtain a detailed history from the parents, and a thorough physical examination is emphasized to identify any characteristic trauma indicative of child abuse or neglect [1]. In the present case, the SCAN team was able to rule that out.

The presence of contact between the needle and dura mater may lead to sensory or motor deficits, CSF leaks which lead to infection or meningitis, and vertebral artery injury [8]. Therefore, surgical removal should be performed as soon as possible, before the appearance of deficit, in order to avoid complications, and to achieve good neurological outcomes [4].

## 4. Conclusion

We present the first report of an incidental IV syringe needle in the cervical spine. Our patient presented with no symptoms or signs and had no postoperative complications. In pediatric patients with a foreign body finding, adequate history taking and investigations are crucial to exclude child abuse and uncommon complications. Migration of foreign bodies to neighboring structures may be considered as a possibility. Identification and surgical removal are key to avoid long-term neurological sequelae even when the patient is asymptomatic. According to the location of the needle, preoperative angiogram and attention to intraoperative CSF leakage muse be considered vital.

## Figures and Tables

**Table 1:** Cases of needles in the cervical spine.

		Clinical data	Examination	Image finding	Surgical complications
1	Silvestro, 2001	A 26-year-old man presented with pain, numbness, and mild hypoesthesia in the right hand. Sewing needle accidently penetrated the cervical spine when the patient fell during a soccer game 5 years earlier.	Normal exam	Needle projecting anteriorly across the right C7–D1 conjugate foramen at the level of the C8 root. The tip of the needle was inside the canal, with the eye of the needle outside.	None
2	Liou, 2007	A 29-year-old man presented with 3-month history of neck pain, bilateral shoulder pain, and sensory disturbance of the left lateral arm. The patient had received an acupuncture-like (*Xiaozendao* acupuncture) procedure, by a nonmedical practitioner, about 3 years ago.	Decreased pain sensation over the upper-lateral area of the left arm. With normal motor function	A 4.8-cm-long metallic needle that tilted toward the tracheal wall at the C2 level running posteriorly and penetrated the epidural space from the left side lamina into the lateral mass of C2 vertebra.	None
3	Anderson, 2007	A middle-aged woman who inserted a sewing needle into her spinal cord in an attempt at performing her own acupuncture. A clean entry point at the midline upper neck.	Decrease in left-arm power (4/5) and altered sensation over the left C5–C8 dermatomes. (Spinal cord injury)	Large sewing needle in the posterior spinal soft tissues between C1 and C2 with the tip within the spinal canal	None
4	Arizumi, 2016	A 47-year-old, presented with discomfort in the posterior region of his neck. He stabbed the posterior region of his neck with a sewing needle 2 years before presentation	Did not reveal an obvious entry point. Normal exam	A 5 cm needle, running posteriorly from between the C2 and C3 spinous processes to the anterior cervical spine and penetrated into the foramen transversarium, in contact with wall of the cervical dura mater and passing near the right vertebral artery.	cerebrospinal fluid (CSF) leakage
5	Persad, 2020	A 16-year-old, presented with neck discomfort after eating a bowl of fish-shaped crackers. Over the next 3 days, she developed odynophagia and hoarseness.	Did not reveal an obvious entry point. Normal exam	A needle-shaped object running anteriorly entering the left C4–5 interspace, passing medial to the vertebral artery with no violation of the vertebral artery.	None
6	Kawamura, 2023	A 37-year-old man presented with neck pain and gait disturbance. While he was self-acupuncturing, an acupuncture needle accidently broke.	Normal exam	Acupuncture needle had entered the cervical canal posteriorly between the C1 and C2 spinous processes penetrating the cervical spinal canal and the dura mater, contacting the cervical spinal cord.	None
7	Present case	A 2-year-old child with an incidental finding of a foreign body in her neck.	Normal exam	A 4 cm needle-shaped object entering posteriorly between the right C7 and T1 interspinous space and ending at the level of C5-C6 interspace, passing medial to the vertebral artery with no violation of the vertebral artery.	None
